# Can role model women in politics help married women reduce suicides? Evidence from a quasi-experimental study in Brazil

**DOI:** 10.3389/fpubh.2024.1513669

**Published:** 2025-01-10

**Authors:** Gabriela Gerote Arvate, Paulo Arvate, Adriano Massuda, Raffael Massuda, Rifat Atun

**Affiliations:** ^1^Faculdade Israelita de Ciências da Saúde Albert Einstein Hospital Israelita Albert Einstein, São Paulo, Brazil; ^2^São Paulo School of Business Administration - Getulio Vargas Foundation, São Paulo, Brazil; ^3^Department of Forensic Medicine and Psychiatry, Federal University of Paraná, Curitiba, Brazil; ^4^Harvard Center for Population and Development Studies, School of Public Health, Harvard University, Cambridge, MA, United States

**Keywords:** gender inequality, social norms, married women’s suicide, role model women, regression discontinuity design

## Abstract

**Background:**

The medical literature has demonstrated that macro-variables and social factors can influence suicide rates. Additionally, social science literature has shown that women in prominent political positions (such as mayors) can influence the behavior of other women. The purpose of our work is to demonstrate that women in such positions reduce suicide rates within a group affected by gender inequality: married women.

**Methodology:**

We use regression discontinuity methodology and quasi-experimental electoral designs (elections with a margin of victory very close to zero) to ensure causal inference between the election of women and suicide rates among married women.

**Principal findings:**

Municipalities that elected women as mayors have 1.33 fewer suicides among married women per 100,000 inhabitants compared to those that elected men as mayors.

**Conclusion:**

These results offer new insights into how empowered women can challenge social norms and improve public health outcomes.

## 1 Introduction

Mental illness is the greatest burden of disease worldwide and accounted for 32.4% of years lived with disability (YLDs) and 13.0% of disability-adjusted life years (DALYs) in 2015 (1). In 2016 there were an estimated 817,000 deaths worldwide caused by suicide (2). According to the World Health Organization, 77.0% of the suicides worldwide occur in low- and middle-income countries (3). While suicide rates have been reducing all over the world this is not the case in the Americas (3). In 2016 mental and neurological disorders, substance use disorders and self-harm (MNSS) in the Americas accounted for 19% of the total number of disability-adjusted life years (4). Despite the very high burden of MNSS, however, median spending by governments in the Americas on mental health was strikingly low, accounting for just 2.4% (IQR 1.3–4.1) of total government spending on health (4). The promotion of mental health and wellbeing is incorporated into Target 3.4 of Sustainable Development Goal 3 (Ensure healthy lives and promote well-being for all at all ages) (5). Target 3.4.2, specifically, aims to reduce suicide mortality rates per 100,000 inhabitants by 30% between 2015 and 2030.

Brazil ranks ninth in the world in terms of the absolute number of deaths from suicide (6). Despite the global agenda for preventing, controlling and reducing suicides and national efforts in Brazil, suicide rates have increased in almost all regions in the country over the last two decades (7, 8). Data from the Brazilian Ministry of Health show that the number of deaths from suicide increased from 19.72 per 100,000 inhabitants in the period 2001–2004 (average of the years) to 21.61 per 100,000 inhabitants in the period 2013–2016 (9). In young, adult and older adult women the suicide rates in Brazil increased between 2000 and 2015 (7). Drug and alcohol poisoning is the most common method of suicide in women in Brazil (10). But suicide is a phenomenon that depends on several factors and can be modified by social interventions. Both population and individual factors interact with these social mechanisms and can reduce the risk of individuals developing suicidal behaviors (11). Thus, there is no single strategy for preventing suicide (12).

The aim of this study is to test the hypothesis that role model women (a macro-political variable) help reduce the number of women suicides. Role model women can be a source of inspiration that changes the behavior of women. We developed this hypothesis by examining three bodies of literature.

First, in different works in economics and business we observe that the existence of a role model women increases the number of women receiving higher salaries in public organizations (13), raises the aspirations and educational attainment of girls (14), and encourages adolescent girls to participate in politics (15). The basis on which the role model woman produces results vis-à-vis the behavior of women is examined in the works of Bandura related to social cognitive theory (16–20). According to social cognitive theory, the existence of women leaders may increase women’s self-esteem and encourage them to change their behavior in historically men-dominated environments. Proactive behavior in an adverse environment can help reduce suicides in women.

Second, we observe in the medical literature that macro-variables may have an influence on suicide rates. Macroeconomic variables (unemployment, recession) are positively correlated with suicide (21–24), while microeconomic variables (policies like a conditional cash transfer program) are negatively correlated with suicide (25).

Third, and also found in medical literature, we observe that social factors affect the risk of suicide (11). Milner et al. showed that there is a reduction in suicide rates in women in countries with increased gender equality (measured by the Gender Gap Index) (26). In gender investigations, these factors are explicit, with men being influenced by financial success and employment (27), while women are influenced by factors that are associated with the family, such as being abused by their husband or by other members of the family, family conflict, or forced marriage (28). In the case of women suicides that are conditioned by social factors, there are studies showing that it is possible to combat these effects by changing the family culture (29–31).

We selected mayoresses as our role model woman through a quasi-electoral municipal experiment to ensure causality between our independent and dependent variables (13, 15, 32). Lee et al. use a quasi-electoral district experiment (two parties—Democratic and Republican—running for one seat) involving the US House of Representatives between 1946 and 1995 to observe if there is a difference in the implemented policies (32).

Our main result shows that municipalities with elected women have 1.33 fewer suicide deaths among married women per 100,000 inhabitants than municipalities with elected men. This population group either “lives” the aspirations of their husbands or suffers family abuse. These findings reinforce the importance of the social, economic and political empowerment of women by way of more extensive public policies that positively affect women’s health.

## 2 Related literature

### 2.1 The role model effect

The basis of Bandura’s (16–20) social cognitive theory is that individuals learn by observing and imitating the behavior of other individuals. This cognitive process is important for minority/subordinate groups because when members of a group observe and imitate their leader, for instance, this can activate the ability of other members who are targeting the same position to overcome barriers. Thus, women leaders are “role models” who bring about a change of behavior in the women who are subordinate in different environments.

There is a series of works in the literature demonstrating that the “role model effect” is present when women are in a leadership position. Arvate et al. (13) showed that the fact of women being elected to become mayors leads to an increase in the number of women who receive higher salaries in public organizations in Brazil as top and middle managers. Mayoresses choose more women as top managers in public organizations and these in turn choose more women as middle managers. A role model woman has an impact on the perception of other women with regard to their own capabilities. The result, however, is not the same in private organizations. Thus, the effect observed depends on the institutional environment (public or private). In the same way, Beaman et al. (14) showed that political seats reserved for women leaders in randomly selected villages in India (a 1993 law) have an influence on the aspirations of adolescent girls regarding their careers and improve their educational achievements. The authors suggest that role model woman determined these results. Finally, Arvate et al. (15) highlighted that women who are elected as mayoresses increase the number of adolescent girls who register to vote in Brazil when compared to the number of adolescent boys (16- and 17-year old adolescents). Voting is mandatory in Brazil for literate individuals between 18 and 70 years old, although it is not mandatory for 16 and 17 year olds, who can choose whether to participate in the election or not. As in the previous works, the authors suggest that the observed result is affected by role model woman.

### 2.2 Macro-variables and their influence on suicide

Reeves et al. (21) showed that the Great Recession (2007) was correlated with an increase in the suicide rate in Europe and Canada. According to these authors, economic shocks produce two factors that increase the suicide rate. First, a recession increases the number of job losses, heightens the risk of depression, and can increase the rate of suicide too. Second, the prospect of returning to work after losing employment can generate a risk of mental health and suicide. In a different methodological way, Glonti et al. (22) chose different works to produce a survey showing how economic crises affect the mental health (including suicide and attempts at suicide) of individuals in different population groups in different countries in Asia, Europe and North America. Unemployment is recurrently associated with problems of mental health, and economic crises affect women more than they do men. Oyesanya et al. (23) also produce a systematic review of the literature dealing with economic recession and suicide showing that there is a positive correlation between these variables in the vast majority of the works they investigated (almost 82%).

Finally, Meda et al. (24) investigated and showed that the correlation between unemployment/GDP *per capita* and suicide is positive in 175 countries (there is no geographical concentration of countries in the sample). The idea is that the increase in unemployment worsens mental health and increases suicide while an increase in GDP *per capita* has the contrary effect.

### 2.3 Social factors and the risk of suicide

Using a sample of 87 countries, Milner et al. (26) showed that gender equality (a social factor) is associated with a reduction in women’s suicide rates. According to these authors, gender equality benefits women because it produces several improvements in women’s social lives thus reducing this kind of mental health-related problem. Likewise, but using a different methodology, Khan et al. (27) interviewed 35 individuals with a history of attempted suicide in the Lower Dir District in Pakistan and showed that gender is an important factor in suicide rates. Although the rate of suicides among men is higher, women attempt suicide more often than men. This difference occurs because women are more likely to choose non-lethal methods (insecticides and drugs) when attempting suicide while and men choose more lethal methods (a firearm). Moreover, men are influenced more by economic factors (unemployment and financial failure) when attempting suicide than women.

Savani et al. (28) chose 15 different works to produce a systematic survey to understand the motivation behind suicides in Central Asia. They identify mental health (depression, alcohol use, and general mental issues), family and community systems (abuse by husband, abuse by others, family conflict, forced marriage, and negative rumors), socio-economic issues (poor education, work conflict, husband’s migration and poverty), services (inadequate care available and support unwilling to help), and environment (climate, exposure to radiation, and exposure to trauma) as (risk) factors associated with suicide. Indeed, the highest incidence of factors observed are abuse by the husband and others (family).

Finally, some works emphasize domestic violence. Dasgupta et al. (29) interviewed 12 educated Asian Indian women who migrated to the USA because of abusive husbands. Colucci and Monstesinos (30) also produce a survey with works of literature indicating the association between violence against women and suicide among immigrants showing that domestic violence against women is the main factor of suicide.

Based on the existing related literature, we decide to examine whether role model women in politics (a macro-level variable), through their behavior and values, inspire married women to adopt similar behaviors, thereby challenging the gender inequality imposed by social norms (particularly within their families) and mitigating social factors that increase the risk of suicide.

Role Model Women in Politics → Women (Married) → Risk of Suicide

↓

Social Norms → Gender Inequality

## 3 Methodology and data

To estimate our results, we used the quasi-experimental gender electoral disputes of mayors in Brazilian municipalities and the regression discontinuity (RD) methodology (33). We classify gender-based electoral disputes for mayoral positions as quasi-experimental because we focus on elections decided by a margin of victory close to zero. In such cases, it was impossible to predict ex-ante the gender of the winners and losers in each municipality. The Regression Discontinuity Design (RDD) methodology enables us to estimate the differences between municipalities with female winners (treated) and male winners (control) in gender-based elections, focusing on cases where the margin of victory is close to zero (our discontinuity).

### 3.1 Gender electoral disputes with a margin of victory/defeat close to zero (a quasi-experimental approach)

We selected municipalities in which a woman is running against a man (for first or second place) for mayor, there is no second round (in municipalities with a second round the vote can be strategic) (34), and the decision of election occurs when the margin of victory of candidates is close to zero. Theoretically, in this type of election, the observable (for example, characteristics of candidates, municipalities, and the electoral year) and non-observable characteristics are similar. As consequence, the decision of victory or defeat of a woman candidate is similar to tossing a coin, which means the election result is like a random event (a quasi-experiment).

### 3.2 Application of the RD methodology

The RD methodology permits estimating the difference of results between the municipalities in which a woman was elected (role model woman) and municipalities in which a man was elected when the margin of victory of elections is close to zero. Its implementation, however, requires different tests. These tests allow us to confidently determine that the effects are caused by the woman leader and not by any feature of the local environment, additional leader characteristics, or any specificities of the electoral process.

To ensure this internal validity, we adopted the following (six) tests (see the description of procedures in Lee & Lemieux combined to Marshall) (35, 36). First, by using a set of histograms of elections with different bins (i.e., the windows used for making the histogram with the number of women victories and defeats: 2, 1, and 0.05%; visualize the result in Appendix Figure A1—left side) and McCrary’s test (2008: it rejects the null hypothesis for the difference in density estimates just to the left and just to the right of the cut-off—zero margin; visualize the result in Appendix Figure A1—right side), we guarantee that there is no electoral manipulation. Second, we verify if the mayor (graduated from secondary school, graduated from college, PT party, PSDB party, DEM party, and MDB party) and municipal characteristics (% of women elected as municipal councilors, % of votes for women as municipal councilors, % of municipal residences with Internet, % of literate individuals in the municipality, per capita municipal GDP, number of vaccines applies, % of women in the municipality, the municipal Theil index, % of rural domiciles in the municipality, the number of families in the cash-transfer program, and the lagged women homicides—the 2000–2004 term) are balanced (visualize the result in Table 1, in the last three columns on the right). Any imbalance coming from the covariates might explain the electoral results. For example, an imbalance in the percentage of women between municipalities could explain the electoral result of an elected woman. Our municipal characteristics follow the standard of other works that have investigated the effect of economic variables on suicide rates (21–24) (the figures showing the balance of the covariates are in Appendix Tables B1–B3).

**Table 1:** Descriptive statistics and RD estimates on covariates.

	Municipalities with an elected men			Municipalities with an elected women			Elected men vs. women—RD estimate—covariates		
	Obs.	Average	SD	Obs.	Average	SD	Obs.	Obs. Effective	coef/s.e.
Women suicides	1,522	5.60	9.39	1,071	5.48	10.64	–	–	–
Men suicides	1,522	23.40	22.97	1,071	22.73	24.16	–	–	–
Women suicides (schooling of between 1 and 3 years)	1,522	5.71	10.72	1,071	5.57	11.04	–	–	–
Women suicides (schooling of between 4 and 7 years)	1,522	6.54	11.54	1,071	6.18	11.02	–	–	–
Women suicides (schooling of between 8 and 11 years)	1,522	3.44	7.63	1,071	3.21	8.43	–	–	–
Women suicides (schooling of more than 12 years)	1,522	0.78	3.06	1,071	1.02	3.94	–	–	–
Women suicides (single)	1,522	2.19	5.35	1,071	2.38	5.93	–	–	–
Women suicides (married)	1,522	1.88	5.59	1,071	1.49	5.30	–	–	–
Women suicides (legally separated)	1,522	0.42	3.80	1,071	0.41	2.80	–	–	–
Women suicides (widow)	1,522	0.50	3.13	1,071	0.37	2.62	–	–	–
Women suicides (adolescents between 15 and 19 years)	1,522	0.48	2.37	1,071	0.55	2.84	–	–	–
Women suicides (adults between 20 and 39 years)	1,522	1.92	5.15	1,071	2.04	5.38	–	–	–
Women suicides (adults between 40 and 69 years)	1,522	2.41	6.16	1,071	2.23	6.74	–	–	–
Women suicides (adults over 70 years)	1,522	0.56	3.51	1,071	0.48	3.36	–	–	–
Women suicides (yellow)	1,522	0.04	3.80	1,071	0.01	0.26	–	–	–
Women suicides (white)	1,522	2.62	7.05	1,071	2.54	7.85	–	–	–
Women suicides (Indian)	1,522	0.11	1.51	1,071	0.16	2.36	–	–	–
Women suicides (multiracial)	1,522	2.35	6.01	1,071	2.22	6.23	–	–	–
Women suicides (black)	1,522	0.25	1.77	1,071	0.32	2.82	–	–	–
Graduated from secondary school	1,541	0.34	0.47	1,090	0.29	0.45	2,631	1,656	−0.0543/0.05559
Graduated from college	1,541	0.41	0.49	1,090	0.61	0.48	2,631	1,750	0.260/0.0549***
PT (Workers Party)	1,541	0.09	0.29	1,090	0.08	0.28	2,631	1,612	−0.0295/0.0389
PSDB (Brazilian Social Democracy Party)	1,541	0.12	0.33	1,090	0.13	0.34	2,631	1,914	−0.00385/0.0336
DEM (Liberal Party)	1,541	0.08	0.28	1,090	0.08	0.28	2,631	1,918	−0.00971/0.0315
MDB (Brazilian Democratic Movement Party)	1,541	0.19	0.39	1,090	0.20	0.40	2,631	1,692	−0.0270/0.0441
% of women elected as municipal councilors	1,540	0.14	0.11	1,090	0.15	0.12	2,630	1,674	0.0204/0.0148
% of votes for women as municipal councilors	1,535	0.17	0.07	1,074	0.17	0.07	2,609	1,666	0.0145/0.00858*
% of municipal residences with the Internet	1,533	0.13	0.10	1,086	0.12	0.10	2,619	1,960	0.00897/0.0103
% of literate individuals in the municipality	1,541	70.33	15.62	1,090	69.88	15.73	2,631	1,589	−0.0128/1.905
Per capita municipal GDP	1,522	3.96	5.76	1,071	3.93	6.73	2,593	1,149	−0.146/0.414
Number of vaccines applied (public vaccine)	328	0.20	0.06	253	0.20	0.05	581	390	0.00670/0.0169
% of women in the municipality	1,471	0.49	0.01	1,028	0.49	0.01	2,499	1,411	−0.000592/0.00202
the municipal theil index	1,522	0.52	0.10	1,071	0.52	0.11	2,593	1,595	−0.00441/0.0127
% of rural domiciles in the municipality	1,463	0.59	0.22	1,030	0.59	0.21	2,493	1,633	0.0370/0.0247
Number of families in the cash-transfer program (PBF)	1,541	2,071.90	3,195.52	1,090	2,247.14	4,576.96	2,631	2,085	1005.5/689.46
Lagged Women homicides (the 2000–2004 term)	1,375	50.48	29.60	968	50.61	29.76	566	404	−5.018/6.345
The 2004 mayoral elections	1,541	0.24	0.43	1,090	0.25	0.43	2,631	2,035	−0.00381/0.0430
The 2008 mayoral elections	1,541	0.29	0.45	1,090	0.26	0.44	2,631	1,712	−0.0947/0.0523*
The 2012 mayoral elections	1,541	0.45	0.49	1,090	0.47	0.49	2,631	1,695	0.113/0.0564**

*Significance at the 10% level, ** at 5% level, and *** at 1% level; Robust standard errors clustered at the municipal level; (1) Bias-corrected RD estimates with robust variance estimator using Calonico et al. (37). The difference, or not, between candidates in the 2008 and 2012 elections does not indicate any advantage for the candidates.

**Figure 1:**
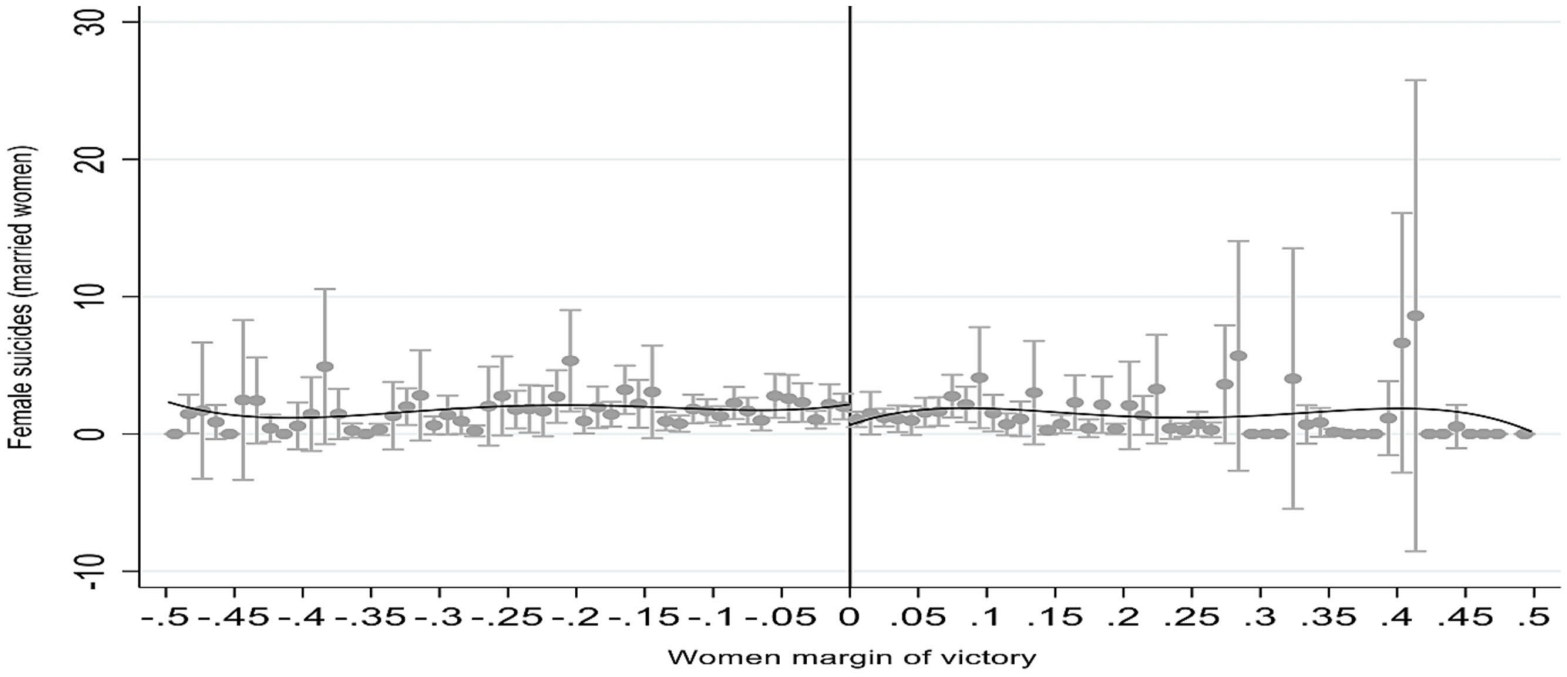
Women leadership and women suicides (married). Positive margin of victory indicates woman victory and negative margin of victory indicates woman defeat (man victory). Source: Authors own.

Our third test verified whether our dependent variables (relative to suicides) do not come from the past, but after the election (our quasi-experiment). For those variables that are statistically significant at the 5% level after the quasi-experiment, we verify if the same quasi-experiment explains what occurred in the previous electoral term (2001/2004; the results are in the same table as the main result; see Table 2, columns 1–5). In the fourth test we produce evidence of our main results with different bandwidths (bandwidth refers to how wide a range of the assignment variable around the cutoff is used to fit the local regression; the choice of bandwidth involves a tradeoff between bias and precision in the estimation: the higher bandwidth is more precise and increases bias, while the lower bandwidth causes the contrary effect); since the RD result is local (Local Average Treatment Effect), the same result with a lower and higher bandwidth enables us to infer also that there is external validity in the results (the results of the estimate of our main variable, women suicides (married) in Table 2, show the same evidence with different bandwidths) (35, 36). Following the application of methodology, in the fifth test we produce evidence of our main result for different polynomials. Elevated polynomials of the assignment variable may contain a bias; the RD estimate is essentially the difference between the weighted average of the dependent variable for treated observations—victory—on one side of the discontinuity and the weighted average of the same dependent variable for control observations—defeat—on the other side of the cutoff. Fitting a high-order polynomial can mean this weighted average is driven by observations that are far away from the cutoff. All our results (see Table 2) were generated with a first-degree polynomial and our main result, women suicides (married), was also generated with a second-degree polynomial. The results are similar. Finally, in the last (sixth) test, we show that the same discontinuity (at the cutoff point) observed in the estimates of the dependent variables is visually observed in figures (inspecting the estimated version is a simple powerful way to visualize the identification strategy). We show the main result (women suicides [married]) in Figure 1.

**Table 2:** Effect of women leadership on women suicides.

Dependent variables:	Backward results (election 2004 and the 2000–2004 term)					Forward results (three elections: 2004, 2008, and 2012; three terms: 2005–2008; 2009–2012; 2013–2016)				
	Coefficient	SE	Effective number of observations	Number of observations	Bandwidth	Coefficient	SE	Effective number of observations	Number of observations	Bandwidth
	(1)	(2)	(3)	(4)	(5)	(6)	(7)	(8)	(9)	(10)
Women suicides	–	–	–	–	–	−1.294	(1.097)	1,487	2,593	0.131
Men suicides	–	–	–	–	–	−3.682	(3.240)	1,398	2,593	0.120
Women suicides (schooling of between 1 and 3 years)	−8.985**	(4.040)	100	234	0.083	−4.048***	(1.398)	1,488	2,593	0.132
Women suicides (schooling of between 4 and 7 years)	–	–	–	–	–	−1.297	(1.310)	1,499	2,593	0.133
Women suicides (schooling of between 8 and 11 years)	–	–	–	–	–	0.619	(1.078)	1,893	2,593	0.191
Women suicides (schooling of more than 12 years)	–	–	–	–	–	0.275	(0.413)	1,846	2,593	0.182
Women suicides (adolescents between 15 and 19 years)	–	–	–	–	–	0.0137	(0.301)	1,523	2,593	0.136
Women suicides (adults between 20 and 39 years)	–	–	–	–	–	−0.252	(0.580)	1,896	2,593	0.191
Women suicides (adults between 40 and 69 years)	–	–	–	–	–	−0.952	(0.796)	1,391	2,593	0.119
Women suicides (adults above 70 years)	–	–	–	–	–	0.0365	(0.247)	1,384	2,593	0.118
Women suicides (single)	–	–	–	–	–	0.0144	(0.614)	1,741	2,593	0.167
Women suicides (married)	−0.605	(2.459)	118	234	0.100	−1.332**	(0.588)	1,299	2,593	0.108
						−2.129**	(1.058)	640	2,593	0.050
						−1.151*	(0.636)	1,628	2,593	0.150
Women suicides (married)—Second-degree polynomial						−1.4576**	(0.651)	1,835	2,593	0,180
Women suicides (legally separated)	–	–	–	–	–	0.335	(0.464)	1,964	2,593	0.206
Women suicides (widow)	–	–	–	–	–	−0.177	(0.287)	1,357	2,593	0.114
Women suicides (yellow)	–	–	–	–	–	−0.0233	(0.0307)	1,274	2,593	0.106
Women suicides (white)	–	–	–	–	–	−0.384	(0.822)	1,469	2,593	0.127
Women suicides (Indian)	−0.0515	(0.0491)	78	234	0.061	0.210*	(0.127)	1,672	2,593	0.157
Women suicides (multiracial)	–	–	–	–	–	−0.674	(0.619)	2,020	2,593	0.216
Women suicides (black)	–	–	–	–	–	−0.212	(0.190)	1,311	2,593	0.110

*Significance at the 10% level, ** at 5% level, and *** at 1% level; Robust standard errors clustered at the municipal level; (1) Bias-corrected RD estimates with robust variance estimator using Calonico et al. (37); “Coefficient” is the estimated coefficient using Calonico’s et al. (37) estimator; “SE” are the standard errors; “Effective number of observations” is the number of observations chosen inside the window; “Number of observations” is the number of observations evaluated for the estimate (corresponds to the number of observations in Table 2); “Bandwidth” is the window of observations used for the estimate. The choice of bandwidth can be different if optimal and it involves a trade-off between bias and precision. Lower bandwidths reduce the bias and increase the precision. Higher bandwidths increase the bias and reduce the precision. All estimates use the optimal bandwidth generated by software produced by Calonico et al. (37) in the STATA software. In our main result (women suicides [married]) our optimal bandwidth is 0.108. We impose a lower bandwidth (0.05) and a higher bandwidth (0.15) and the results are similar. (2) We show the result of lagged dependent variable to those variables in which the effect of women leadership on suicides is significant (columns 6–10) because the effect can be previous (columns 1–5). The non-significant result on lagged dependent variable indicates that the effect after women leadership is not previous. (3) All results were estimate with the first-degree polynomial.

All non-parametric estimates were calculated using the procedures developed by Calonico et al. for the Stata (StataCorp. 2015. Stata Statistical Software: Release 14) (37). All standard errors are clustered at the municipal level.

### 3.3 Data source

We use four different national public databases with secondary data. First, the electoral information comes from the Superior Electoral Court (the number of votes for each candidate, gender, schooling, parties, the percentage of women elected to be a municipal councilor, and the percentage of votes for women on the municipal council) for three mayoral elections (15): 2004, 2008, and 2012. Periods which we could have more information about different variables used here. Mayors are an important political position in sub-national government, with each mayor being elected for a fixed term of 4 years.

Second, information about suicide deaths (gender, age, schooling, marital status, and race), vaccinations, and homicides (women) comes from the Ministry of Health (*Sistema de Informação sobre Mortalidade*: SIM; homicides and suicides are in line with the classification of the WHO; 10th Edition of the International Classification of Diseases, ICD-10). We measured the average for four terms; one term prior to our first quasi-experiment (2001–2004) and three terms after each election: 2005–2008, 2009–2012, 2013–2016.

Third, data from the 2000 and 2010 census (the percentage of municipal residences connected to the Internet, the percentage of literate individuals in the municipality, per capita municipal Gross Domestic Product [GDP]), the percentage of women in the municipality, the municipal Theil index, and the percentage of rural domiciles in the municipality. The (*Fundação Instituto Brasileiro de Geografia e Estatística*, *FIBGE*) is responsible for the Brazilian census. Data from the 2000 census were used for the 2005–2008 period, and data from the 2010 census were used for two terms: 2009–2012, and 2013–2016. All the primary data are available online on the site of each of the institutions mentioned. We use data from the 2000 census for the 2004–2008 period, the 2007 population census for the 2009–2014 period, and the 2010 census for the 2013–206 period.

Fourth, for the number of families benefiting from the cash-transfer program (*Programa Bolsa Familia* [PBF]) data were obtained from the Ministry of Social Development’s database. We measured the average for three terms since this variable was only used as a covariate: 2005–2008, 2009–2012, and 2013–2016.

### 3.4 Institutional background

Based on the constitutional principles of universality, comprehensiveness, decentralization and social participation, the Unified Health System (*Sistema Unico de Saude*—SUS) altered health system governance and changed the model used for providing healthcare in Brazil (38, 39).

In the early 1990s when the SUS was being set up, federal funds and responsibilities were decentralized to state and municipal governments. To encourage the implementation of national health priorities by municipalities, the Ministry of Health then established conditional transfers (40). For example, financial incentives were created for primary care and channeled to municipalities to adopt the use of Family Health Teams (*Equipes de Saude da Familia*—ESF) (41). For mental health, following the 10.216 Psychiatric Reform law, which was approved in 2001, a similar mechanism was established encouraging the substitution of large-scale psychiatric hospitals by community-based mental health centers, called Psychosocial Support Centers (*Centro de Apoio Psicossocial*—CAPS) (42). Municipal governments, therefore, assumed a leading political role in scaling up health care services in the SUS at the local level (43).

### 3.5 Data analysis

Table 1 shows the descriptive statistics of the variables that were used in our empirical analysis.

We show the number of observations, the average, and the standard deviation for municipalities that elected men and for municipalities that elected women. Unlike the dependent variables, we show the balancing of the covariates (the last three columns in the table). We show the total number of observations and the effective number of observations (those municipalities in which the software chooses to estimate the RD estimate; municipalities with a margin of victory/defeat close to zero). The municipalities are well-balanced in terms of the characteristics of the politicians and municipalities.

Nothing interferes with the municipal election for mayor. The exception at the 5% level has to do with mayors who are college graduates. Given the non-statistical result for mayors who are secondary school (balanced) graduates, we do not interpret schooling as being an electoral advantage for women. We only observe that we have more women winners than men winners when both candidates have completed their higher education. Baltrunaite et al. showed evidence that elected men have lower levels of education than elected women in Italy (44). A specific study is necessary to understand this result, and this lies outside the scope of our investigation here. Studying the paper by Lee et al., we see that the percentage of the black population was the only unbalanced covariate in a set of covariates (32). This study only used secondary data from the public domain.

**Figure 2:**
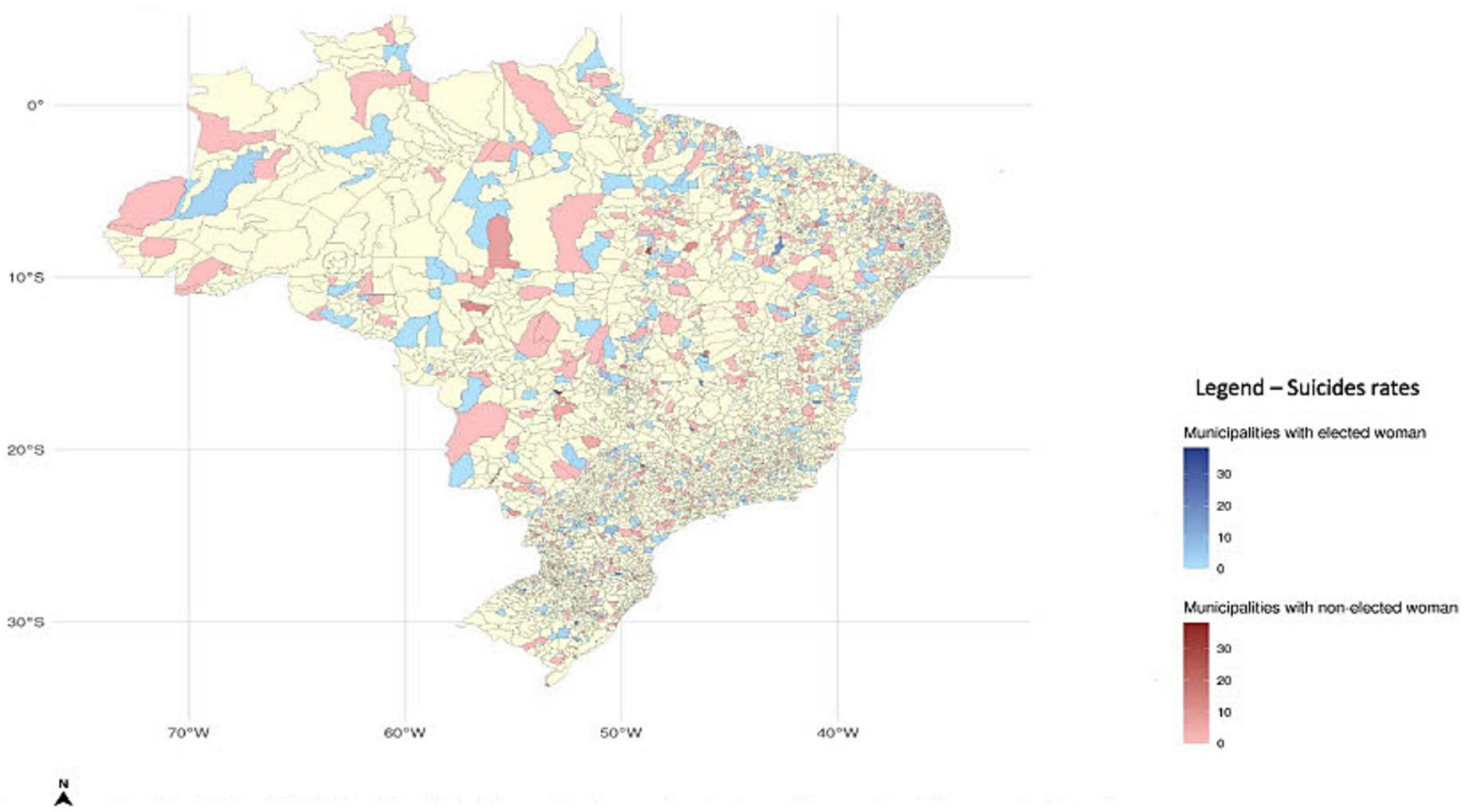
Distribution of municipalities used in the quasi-electoral experiment (the 2012 election) in Brazil and their levels of suicide of married women (in the 2013–2016 term). (North); Scale: 1: 100,000; Municipalities with electoral mixed-gender running; Source: Authors own.

Note that both the women and men suicide rates in these municipalities are above the national average. The distribution in Brazil of the municipalities we used in our quasi-experiment (the 2012 mixed-gender election) and the suicide levels of married women in the 2013–2016 term can be seen in the map of Brazil (Figure 2). The blue municipalities indicate those in which women were elected as mayor. The red municipalities, on the other hand, indicate those in which men were elected as mayor. Darker colors reflect a higher suicide rate and lighter colors reflect a lower suicide rate (other terms are in Appendix Figures C1, C2).

## 4 Results

Table 2 presents the results of our dependent variables (suicide variables by gender—men and women, schooling, marital status, and race).

There are two groups of results in this table. The first group (backward results: columns 1–5) shows the results considering the 2001–2004 electoral term. We show only results for those variables that are statistically significant after the quasi-experiment (2004, 2008, and 2012 elections) to observe if this effect of the variables is not previous. The second group (forward results: columns 6–10) shows the results considering the 2005–2008, 2009–2012, and 2013–2016 electoral terms. Each group contains the same information: the estimated coefficient; the robust clustered standard error at the municipal level; the effective number of observations (used by the software to estimate close to zero margins); the number of observations (municipalities); and the bandwidths (they can be “optimal,” generated by the software, and defined by us as robust).

The forward results reveal that a role model women (mayoress) leads to 1.332 (*p* < 0.05) fewer deaths by suicide among married women per 100,000 inhabitants (optimal bandwidth:0.108) when compared with municipalities that are governed by mayors (men).

We investigated the same effect on other dependent variables (robustness) to show that our result occurs only for one group of the population: married women. We have weak evidence (at the 10% level) that the same role model woman leads to 4.048 (*p* < 0.10) fewer suicide deaths per 100,00 inhabitants in women with low levels of schooling (between 1 and 3 years) but increases the suicide rate by 0.210 (*p* < 0.10) for Brazilian indigenous women, always compared with municipalities that are governed by mayors. The other results (suicides of women with different schooling, age, a different marital status like widow or single, and of a different race) in municipalities are non-significant. We are left, therefore, with one result: the suicide of married women.

Looking at the backward results, we also observe that the result of suicide among women with low levels of schooling (at the 10% level) is not after our quasi-experiment given the significance observed with this variable in the past (−8.985; *p* < 0.05). We do not observe the same significance for the suicides of married women.

Compared with men in the same position, our role model woman (mayoresses), therefore, reduces suicide death rates among married women. The impact is important since women suicide rates in the municipalities in our sample are around five suicides per 100,000 inhabitants.

Figure 1 shows the results of our quasi-experiment with regard to the suicides of married women.

The cut-off (the vertical line in Figure 1) marks a margin of victory equal to zero. A positive margin of victory represents the victory of women, and a negative margin of victory represents the defeat of women (victory of men). Each point represents the average result in each bin (with a confidence interval of 99%). The estimate uses these points. We have two estimates: one for points to the right of the cut-off and the other for points to the left of the cut-off.

Important here are the points around the cut-off; our estimated result is a Local Average Treatment Effect (LATE). Results far from the cut-off point are less homogenous (i.e., the different characteristics of the municipalities can affect the result: remember that our covariates are balanced close to the cut-off: a margin of victory close to zero). Figure 1 results and estimated results (Table 2) are very similar: the difference between municipalities is around 1 per 100,000 inhabitants at the cutoff (comparing suicide among married women in municipalities in which women won vs. municipalities in which men won).

Because of the methodology used here (RD) we believe that our result is a causal relationship between the variables. Re-emphasizing the point; the woman leader is defined by a quasi-experiment (margin of victory/defeat close to zero) and we compare municipalities in these circumstances. However, looking at the distribution of municipalities on the map presented in Figure 2 (the municipalities used in our quasi-experiment), it is clear that we have included all regions in the country and Brazil is quite different in its regions in terms of population and economic reality. The Southeast Region is the most populous and the richest, and the North and Northeast Regions are less populous and the poorest.

## 5 Discussion with related literature, limitations, policy and future research, and perspective

### 5.1 Discussion with related literature

Role model women (mayoresses) have the power to change the behavior of other women because they demonstrate that adverse situations can be overcome (see Table 1: around 15% of the elected councilors are women). In the 2000s, mayoresses entered the men terrain of politics showing that they are capable of leading and winning. Their example can be seen as an incentive to other women living in an adverse environment. The medical literature has shown that the man domestic culture is one of the cultural factors associated with women suicides (28–31). Women’s aspirations are subjugated, and they “live” the aspirations of their husbands or suffer family abuse (from either their own husband, or from other members of the family). Reeves & Stuckler show the importance of egalitarian gender norms (measured through economic and political indicators of the gap between men and women) on suicides in European countries (45). Role model woman can be the trigger that breaks an environment that drives many women to suicide.

The macroeconomic effects of recession and unemployment increase suicide rates, although a Brazilian policy of conditional cash-transfers has reduced them (25). Like the positive correlation that exists between the microeconomic variables of the policy (the conditional cash-transfer program) and suicide (11), we show the positive macro-political effect that leads to a reduction in suicides among important victims of social construction: married women.

Unlike most previous studies that examine the cultural and economic impacts on female behavior, our article highlights an underexplored political dimension: the transformative role of female leaders, such as mayors, in reshaping social norms. While prior research has identified the influence of gender norms on suicidal behavior (45), our analysis uniquely demonstrates how female mayors can serve as direct catalysts for reducing suicides among married women—a group particularly susceptible to cultural subjugation and domestic violence. Moreover, while economic policies like Brazil’s conditional cash transfer program have been acknowledged for their protective effects on suicide rates (11, 25), we contribute a macro-political perspective by showing how role model women can redefine social expectations and foster a more egalitarian and supportive environment for women.

### 5.2 Limitations

The study has two limitations. First, the strong internal validity of the RD design is counterbalanced by poor external validity. In order to overcome this major limitation, we highlighted in Figure 2 that the municipalities used in our investigation come from different parts of the country. The regions of the country are different in terms of their populations (the Southeast Region is the most populous and the richest while the North and Northeast Regions are less populous and the poorest) Second, our suicide deaths variable was built with information from the Ministry of Health. It would be interesting to compare the results of this variable with data obtained from the police departments in each region. Unfortunately, we do not have access to the data held by the police for the whole country.

### 5.3 Policy and future research

Notwithstanding its limitations, our finding for the first time of the positive effect of role model women on suicide rates among married women has important implications for policy makers in that they should focus on the broader global determinants of health that go beyond the social determinants at the individual level. Moreover, further research could explore how empowered women can also enact policies—such as those promoting education and economic empowerment—that may influence suicide rates among other groups of women. Investigating alternative mechanisms that address gender inequality would also be valuable.

Finally, although our quasi-experimental design focused on municipalities balanced in terms of per capita income, and our main exercise demonstrated that the suicide rate is not significantly influenced in racial, educational, or marital status (except for married women), we recognize that income plays a pivotal role in mitigating gender inequalities, as noted in the literature. Future research could further explore how women across different income levels might respond differently. For instance, while our findings did not observe a significant effect on the suicide (average) rates of single women, it is possible that single women with high income and those with low income experience distinct outcomes. Similarly, disparities may exist between other groups, such as rich married women and poor single women, depending on income definitions. Investigating these nuances could provide a deeper understanding of the complex interplay between socioeconomic factors and suicide rates.

### 5.4 Perspective

Furthermore, the findings reinforce the importance of the social, economic and political empowerment of women through broader public policies for positively affecting women’s health (46). Our study also provides new evidence that underpins the importance of a comprehensive and integral multi-sectoral approach to health, as envisaged in the United Nations Sustainable Development Goals (47).

## 6 Conclusion

This work is important in that it highlights how women of importance/role models (mayoresses) help/encourage other women to find ways of overcoming family arrangements that may subjugate them, which thus deters them from committing suicide (married women). Other works have already demonstrated this importance in other areas in emerging countries as women occupying positions in public organizations, improvements in the aspirations of adolescent girls and their educational attainments, and the increased participation of adolescent girls in politics. Other works in medical literature have shown that women commit suicide because of a family arrangement, but no work has shown how a social macro-variable (the gender of the mayor) might intervene and produce results in this family arrangement that would help women.

This is one work among others that show the importance of the social, economic and political empowerment of women by way of broader public policies that have a positive effect on women’s health.

## Data Availability

The datasets presented in this study can be found in online repositories. The names of the repository/repositories and accession number(s) can be found in the article/Supplementary material.
